# Hidden Threat: Incidental Finding of Pancreatic Body Solid Pseudopapillary Tumor During Bariatric Evaluation With an Open Central Pancreatectomy Resolution

**DOI:** 10.7759/cureus.60116

**Published:** 2024-05-11

**Authors:** Alejandro Martinez-Esteban, Kevin J Fuentes-Calvo, Natalia M Barron-Cervantes, Alejandra Flores, Javier Ramos-Aranda, Luis F Arias-Ruiz, Carlos Chan

**Affiliations:** 1 General and Gastrointestinal Surgery Service, Fundación Clínica Medica Sur, Mexico City, MEX; 2 Hepato-Pancreato-Biliary Surgery, Fundación Clínica Medica Sur, Mexico City, MEX; 3 Anatomic Pathology, Fundación Clínica Medica Sur, Mexico City, MEX

**Keywords:** pancreaticojejunal anastomosis, modified blumgart-type, gastric bypass, pancreatic resection, central pancreatectomy, pseudopapillary solid tumor

## Abstract

Incidentalomas, or tumors found incidentally, are very common. However, pancreatic tumors are usually not found as incidentalomas. To date, these tumors represent a diagnostic and therapeutic challenge, since the risks and benefits associated with surgeries that can be performed to remove these tumors must be evaluated due to perioperative complications. It is vitally important to always carry out a correct approach that includes a histopathological study to allow timely identification of tumors that require surgical management or other preoperative treatment, such as chemotherapy or radiotherapy. The majority of these tumors are benign cystic tumors; however, there are cases, like the one presented here, where the tumor turns out to be a solid pseudopapillary tumor (SPT) that requires a different diagnostic and surgical approach. Also, in this case, the importance of evaluating the patient's general health status is highlighted to determine whether or not the required surgery can be performed at that moment or if any prior intervention is required. This case report talks about a patient in whom an incidental pancreatic tumor was found and how its management was carried out from diagnosis to the postoperative period.

## Introduction

Incidentalomas are tumors that are found during image studies that were solicited for another situation, meaning no clinical presentation or laboratory finding suggested the presence of that tumor. These tumors are the result of the evolution of diagnostic techniques that allow for more clinicians to have access to many noninvasive imaging modalities and thus increasing the number of incidental findings that may not be suspected otherwise, representing a new diagnostic challenge for all clinicians. The main challenge is being able to distinguish between the vast majority of benign tumors that will not need any more diagnostic approaches and the malignant or hormone-secreting tumors that will require further therapy [1]. Pancreatic tumors are more frequently presented nowadays; associated with the increased use of high-quality cross-sectional imaging, the most common imaging study by which these are detected is abdominal computed tomography (CT) scan. These tumors involve a wide spectrum of pathologies, from benign simple cysts to neoplasias, which is why it is extremely important to characterize the lesion. The first thing needed is to search for any specific syndrome associated with a hormone overproduction, which may guide the diagnostic approach toward a neuroendocrine tumor (pNET). If asymptomatic, biopsy should be performed [2].

Solid pseudopapillary tumors (SPTs) are pancreatic tumors of low malignant potential with poorly defined pathogenesis, which occurs most frequently in young women incidentally as a solid or cystic tumor in the body or neck of the pancreas; in general, it represents 1%-2% of pancreatic tumors, was described by V. K. Frantz in 1959, and was incorporated into the World Health Organization (WHO) classification of tumors in 1996 [3]. In general, these tumors present with nonspecific abdominal complaints until they start growing and invading locally adjacent organs, which is why they are mostly diagnosed lately and are associated with a poor prognosis [4]. Surgical resection is the only curative treatment for these patients, with pancreaticoduodenectomy being the most commonly performed because of the local extension at diagnosis [4]. There are a few cases depicted in literature where an early diagnosis allows for a smaller resection to be made, such as a central pancreatectomy, thus allowing the patient to keep more pancreatic tissue and improving the overall life quality, such as in the case presented in this report. We present the case of a 37-year-old female patient who presented to the outpatient clinic with an abdominal ultrasound (US) report that incidentally described an epithelial pancreatic neoplasm compatible with an SPT in a completely asymptomatic previously healthy woman that was being approached for bariatric surgery in a first level private surgical center in Mexico City. This case is presented in order to further expand the knowledge about incidental pancreatic tumor and SPT, as well as highlighting the importance of a close follow-up and an early surgical and oncological management.

## Case presentation

A 37-year-old Latin American female presented to the outpatient clinic with an abdominal ultrasound (US) report that incidentally described as a solid pancreatic neck or body tumor. It is important to mention that the patient was completely asymptomatic, with no data that suggested that diagnosis, this was an image study that was requested as part of her preoperative approach for bariatric surgery. Upon her first consultation with the surgeon, a pancreatic biopsy guided by endoscopic ultrasound (EUS) was requested, which reported an epithelial neoplasm compatible with an SPT in addition to an immunohistochemistry report of positive beta-catenin, positive synaptophysin, and positive transcription factor enhancer 3 (TFE-3) (EP285) and negative chromogranin. As this biopsy was taken at another outpatient clinic, the patient only presented the report and no images were presented. Because of the obesity presented by the patient, the decision was made to remain expectant due to being overweight, which relatively contraindicated surgery, and weight reduction was recommended for subsequent surgical management. After the consultation, the preoperative evaluation continued and the patient was taken to surgery weeks later where a gastric sleeve was performed. In the first six months, a weight reduction of almost 30 kg was achieved, so the patient went back to the outpatient clinic again for evaluation by the hepato-pancreato-biliary surgery team. During her next consultation, an abdominal computed tomography (CT) scan was requested, which reported an infiltrative process in the pancreas or pseudopapillary tumor. This was also taken at the other outpatient clinic and no images were presented, only the final report. Upon physical examination, the patient presented was completely asymptomatic, and no abdominal pain nor palpable abdominal masses were presented and Courvoisier sign was absent. Laboratory tests were requested, in which slightly leukopenia and a negative carbohydrate antigen 19-9 (CA 19-9) were reported, everything else was within normal limits (WNL) (Table 1).

**Table 1:** Laboratory exams. Complete blood count (CBC), relevant data from blood chemistry (BC), hepatic profile, pancreatic enzymes, CA 19-9, and coagulation times. BUN: blood urea nitrogen, INR: international normalized ratio.

Parameter	Value	Reference values
Hemoglobin	13.6 g/dL	12-18 g/dL
Hematocrit	40.9%	36%-48%
Platelets	262 x 10^3^/uL	150-450 x 10^3^/uL
Leukocyte count	3.54 x 10^3^/uL	4.5-11 x 10^3^/uL
Absolute neutrophiles	10.7 x 10^3^/uL	2.5-7 x 10^3^/uL
Serum glucose	79.5 mg/dL	70-100 mg/dL
BUN	10.7 mg/dL	7-20 mg/dL
Serum creatinine	0.73 mg/dL	0.6-1.1 mg/dL
Serum sodium	141.2 mEq/L	135-145 mEq/L
Serum potassium	4.6 mEq/L	3.6-5.2 mEq/L
Serum chloride	107 mEq/L	96-106 mEq/L
Serum calcium	9.4 mg/dL	8.5-10.5 mg/dL
Serum phosphorus	3.4 mg/dL	2.8-4.5 mg/dL
Serum magnesium	2.0 mg/dL	1.7-2.2 mg/dL
Total serum proteins	6.4 g/dL	6-8 g/dL
Albumin	4.31 g/dL	3.4-5.4 g/dL
Total bilirubin	1.09 mg/dL	1-1.2 mg/dL
Indirect bilirubin	0.77 mg/dL	0.2-1.2 mg/dL
Direct bilirubin	0.32 mg/dL	0-0.35 mg/dL
Glutamic pyruvic transaminase (GPT)	13.9 U/L	4-36 U/L
Glutamic-oxaloacetic transaminase (GOT)	12.8 U/L	5-40 U/L
Amylase	64 U/L	30-110 U/L
Lipase	98 U/L	0-160 U/L
Carbohydrate antigen 199 (CA 19-9)	Negative	Negative
Prothrombin time (PT)	10.6 seconds	11-13.5 seconds
INR	0.99	1-1.1
Partial thromboplastin time (PTT)	26.3 seconds	25-35 seconds

**Figure 1:**
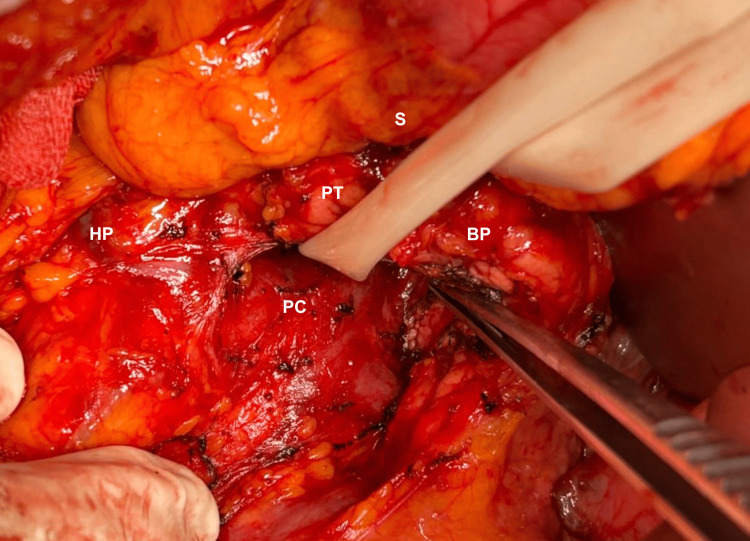
Pancreatic tumor dissection. Surgical dissection involving the stomach, body of pancreas, head of the pancreas, and portomesenteric confluence. S: stomach, PT: pancreatic tumor, BP: body of the pancreas, HP: head of the pancreas, PC: portomesenteric confluence.

During that consult, the decision was made to perform the resection surgery and it was scheduled. One week after that, an exploratory laparotomy and an open central pancreatectomy were performed. First, a midline supra- and infraumbilical incision were mad. Subsequently, the short gastric vessels were ligated to expose the body of the pancreas and pylorus; a superior and inferior peripancreatic dissection was performed with monopolar and harmonic dissector at the level of the neck and body, retrogastric dissection, periportal and hepatic artery lymphadenectomy. During the dissection, a 5-cm solid tumor with a cystic component at the body of the pancreas was seen; the pancreas was soft, and a pancreatic duct of 3 mm was identified (Figure 1).

**Figure 2:**
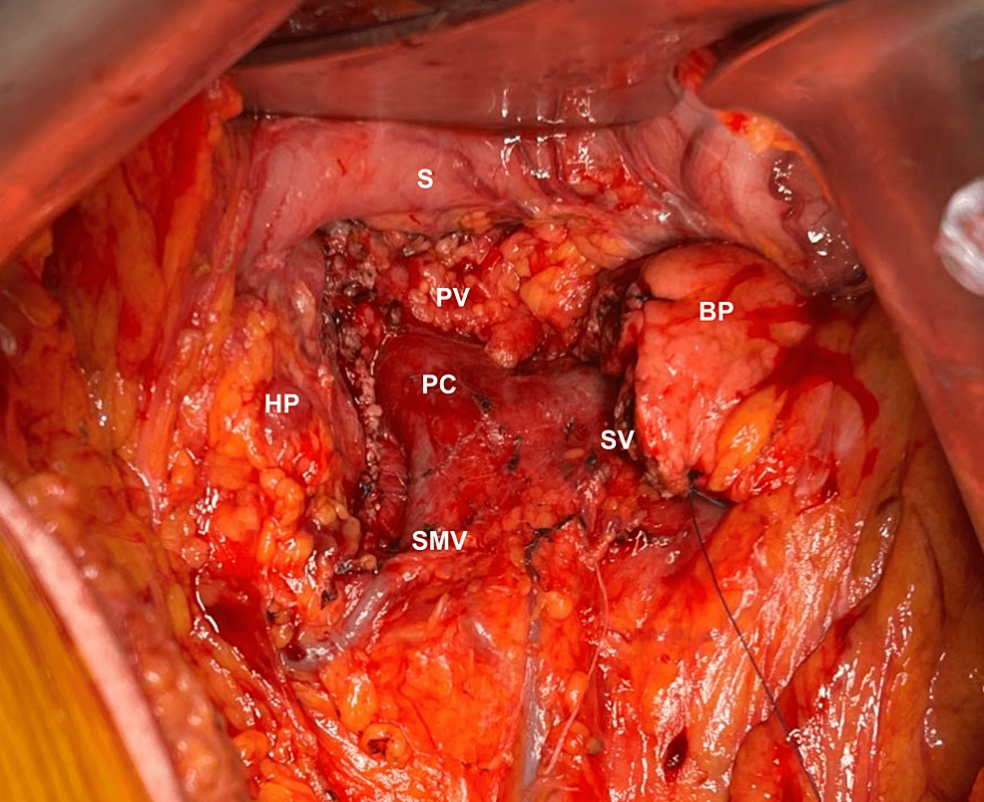
Pancreas after tumor resection. Pancreas remnant including head and body after central pancreatectomy. S: stomach, BP: body of the pancreas, HP: head of the pancreas, PC: portomesenteric confluence, PV: portal vein, SV: splenic vein, SMV: superior mesenteric vein.

Finally, the distal pancreatic division was performed at the level of the body of the pancreas with scalpel and monopolar energy to avoid damage of the remanent pancreatic duct and at the proximal margin with a vascular-sealer 60 mm linear stapler (Figure 2).

**Figure 3:**
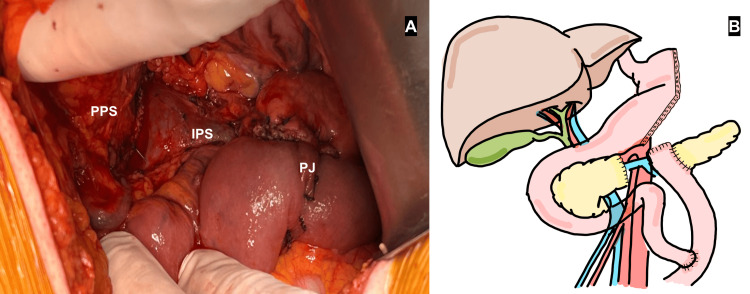
Blumbart’s modified pancreaticojejunostomy. A: Picture taken from surgery showing the Blumbart’s modified duct-to-mucosa pancreaticojejunostomy (PJ). B: illustration depicting the outcome of the surgical procedure, image credits to Natalia M. Barron-Cervantes. PPS: proximal pancreatic stump, IPS: intrapancreatic space.

In order to re-establish pancreatic transit to the gastrointestinal tract, a modified Blumgart-type end-to-side transmesocolic duct-to-mucosa pancreaticojejunal anastomosis was performed with a 5 fr external pancreatic stent tunneled with the Witzel technique (Figures 3A, 3B). To finish the surgery, lavage and drainage of the abdominal cavity was performed with placement of a perianastomotic Jackson-Pratt drain and the skin was closed with surgical staples.

**Figure 4:**
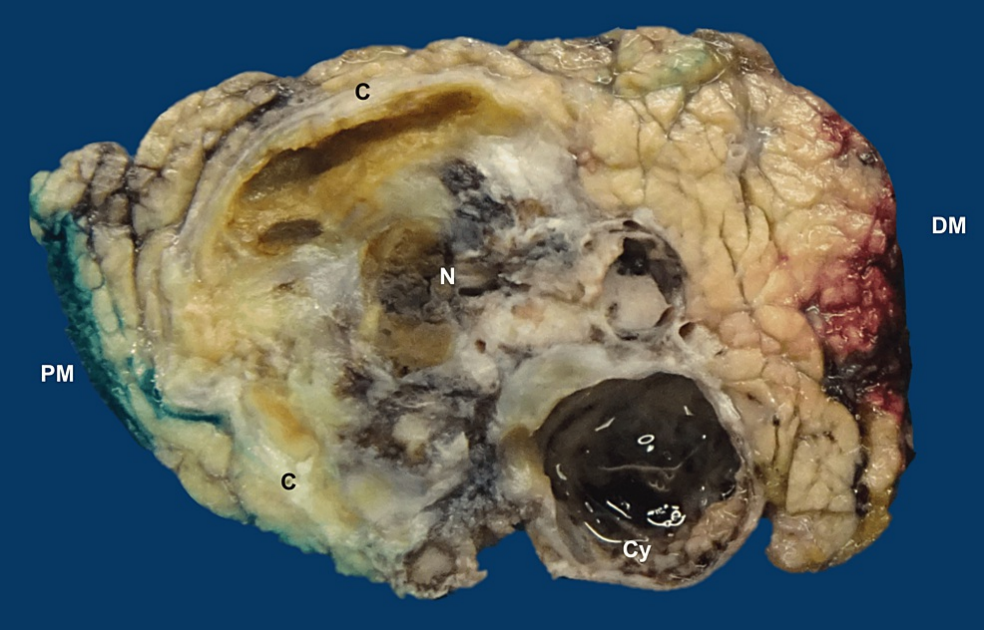
Gross examination of the pancreatic tumor. The tumor was well demarcated by a calcified fibrous capsule (C). It was pale brown, soft, with cystic (Cy), hemorrhagic and necrotic (N) areas. Proximal (PM) and distal (DM) margins were macroscopically negative for the tumor.

**Figure 5:**
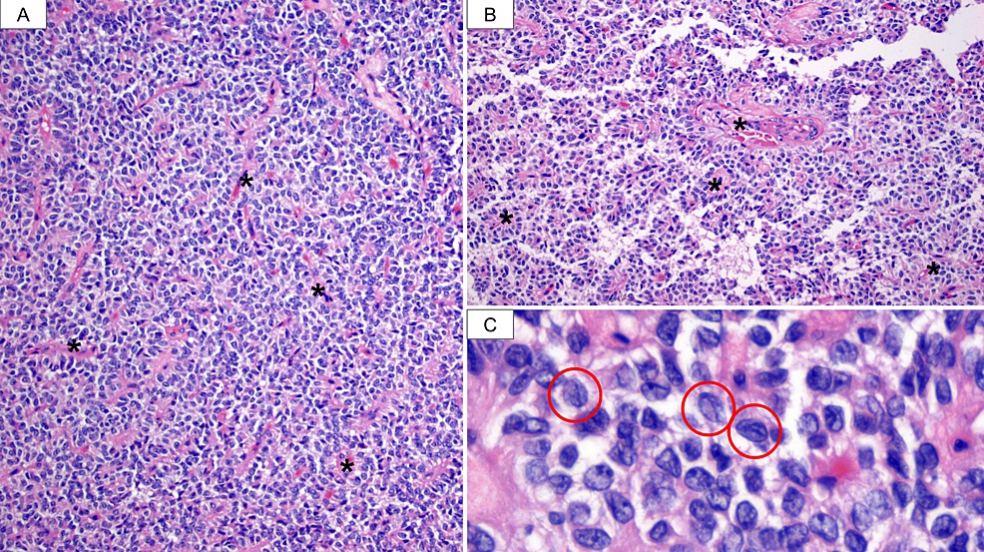
Histopathological study. A: Sections stained with hematoxylin and eosin showed solid pattern features discohesive monomorphic cells that are densely packed and adhere to fibrovascular stalks (*). B: As these cells detach from the stalks (*), they form structures resembling papillae (pseudopapillae) or rosettes. C: Additionally, the neoplastic cells display distinctive longitudinal grooves, highlighted by red circles, imparting a coffee bean-like appearance to the cells.

**Figure 6:**
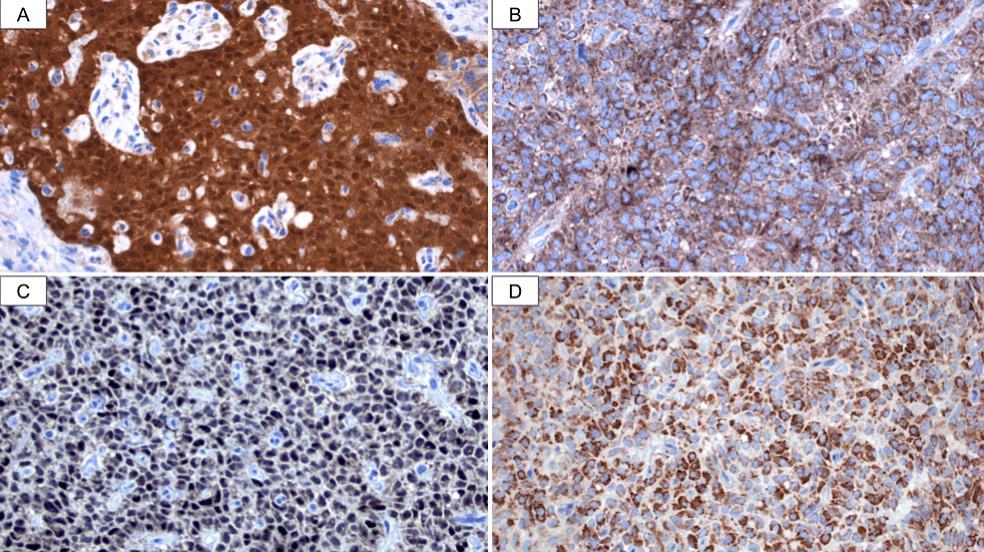
Immunohistochemical study. The neoplasm was positive for (A) β-catenin, nucleus and cytoplasm, (B) CD10, (C) progesterone receptors, and (D) synaptophysin.

On gross examination, the tumor measured 4.0 cm and was confined to the pancreatic margin (Figure 4). Microscopically, the tumor consisted of monomorphic, small, round to cuboidal cells arranged in a solid pattern with abundant small fibrovascular stalks. Some areas showed poorly cohesive cells detaching from the fibrovascular stalks, forming pseudopapillae. The tumor also exhibited areas of fibrosis, hemorrhage, and dystrophic calcification. The cells featured oval nuclei with irregular contours and longitudinal grooves (Figures 5A-5C). The neoplasm tested positive for β-catenin, progesterone receptors, cluster of differentiation 10 (CD10), CD99, chromogranin A, and synaptophysin (Figures 6A-6D). The final diagnosis was that of a solid pseudopapillary tumor. All surgical margins were negative for malignancy.

**Figure 7:**
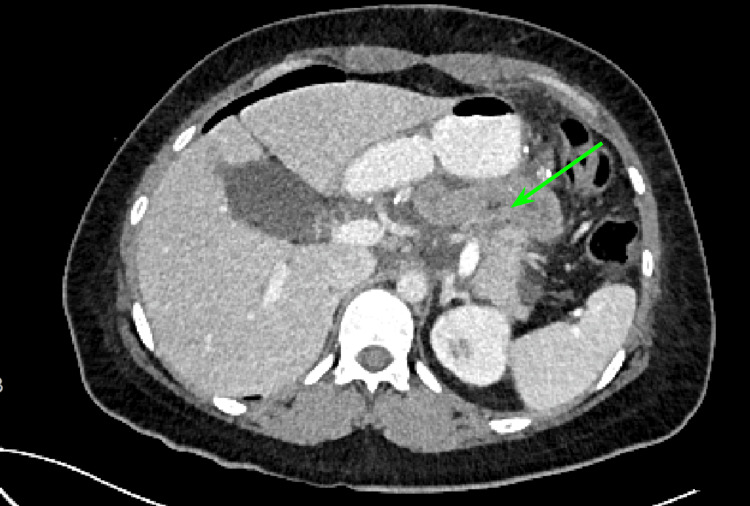
Abdominal CT scan. Edema and stenosis of the jejunum-jejunum anastomosis (green arrow).

On her first postoperative day, the patient was maintained on nothing per oral (NPO), and the results of the amylase in the drainage fluid were collected and found to be elevated at 21,475 U/L at postoperative day (POD) 1 obtaining a maximum of 100,000 U/L at POD 3 as well as a serum lipase of 517 U/L at POD3 thus establishing the diagnosis of a postoperative biochemical leak and post-pancreatectomy acute pancreatitis. During the first week of hospitalization, she began to present nausea and emesis with biliary content and abdominal distension, but the patient reported that she did expel gastrointestinal gas. Due to the clinical manifestations, an NG tube was placed and a double contrast abdominal CT scan was requested. This showed edema and stenosis of the jejunojejunal anastomosis (Figure 7).

Based on the diagnosis given in the abdominal CT scan, it was decided to perform an enteroscopy. During this procedure, gentle dilatation was performed with the endoscope and the patency of the anastomosis was checked with adequate passage of the enteroscope without any eventuality, which was attributed to postoperative edema at the level of the anastomosis and did not require any other specific management. After these procedures, that week he began to have bowel movements without any complications. The patient had a favorable post-surgical evolution; during his stay, he remained hemodynamically stable, without fever and with improvement in symptoms, with adequate tolerance to the oral route and adequate urinary volumes; reason for which it was decided to discharge from hospital with the abdominal drain and an open external pancreatic stent. She is currently asymptomatic, with no other post-surgical complications.

## Discussion

With the advances in modern technology, noninvasive imaging studies use has been constantly increasing in the last decade, specially with CT scans and magnetic resonance imaging (MRI). This trend has resulted in an increase in incidental mass findings, also known as incidentalomas. By definition, an incidentaloma should be detected during abdominal imaging that was indicated for any other reason rather than the diagnostic suspicion of pancreatic disease. The overall prevalence has been as high as 6% to 23% of all abdominal imaging studies present an asymptomatic neoplastic lesion, from which up to 50% are malignant and up to 47% are considered potentially malignant or premalignant [5]. Pancreatic incidentalomas involve a wide group of pathologies that go from benign to malignant and from cystic to solid lesions. Something very important to mention is that cystic pancreatic incidentalomas are the most common, and a number of guidelines that present their corresponding management have been issued throughout the years. However, solid pancreatic incidentalomas remain a diagnostic challenge for clinicians around the world [6]. This diagnosis can contribute to early diagnosis that allows a timely treatment; however, sometimes it can result in inadequate management, overtreatment, and unneeded mortality and morbidity. Therefore, pancreatic incidentalomas are one of the biggest diagnostic challenges presented nowadays [6]. In literature presented, most of the pancreatic incidentalomas are malignant tumors or tumors with a malignant potential; therefore, an endoscopic ultrasound (US) with a biopsy should be performed in order to have a final diagnosis for that mass presented. Almost half of all solid pancreatic incidentalomas were ultimately diagnosed as intraductal papillary mucinous (IPMT) tumors. The advantage presented in these cases is that since they have been diagnosed very early; in most cases, they are noninvasive tumors that can still be treated surgically and in turn are associated with a better prognosis [7].

SPTs were first reported by Dr. Franz in 1959. However, it has been described by multiple names throughout history until 1996 when the WHO first classified this group of tumors as SPT. Incidence goes from 0.1% to 2.7% of all pancreatic tumors and 5% to 12% of all pancreatic cystic tumors that classically present in women in their second decade of life, predominantly seen in African American and Asian communities [8]. They represent a diagnostic challenge associated with a poor prognosis as they initially manifest with nonspecific abdominal complaints, such as nausea, discomfort, and distension. When they grow, this is the reason why they are large tumors at the time of diagnosis, usually going from 6 to 30 cm, and are associated with invasion to bile ducts or even tumor rupture presenting with hemoperitoneum [4]. Laboratory findings are not useful in these cases as it is uncommon for patients to present elevation in pancreatic enzymes and they tend to present with normal serum levels of alpha protein (AFP), carcinoembryonic antigen (CEA), CA19-9, and cancer antigen 125 (CA-125) [9], as shown in this case. When suspected, a noninvasive image study must be performed. SPTs are slightly more common in the pancreatic tail and can be seen in abdominal CT scans as well-defined masses presenting low-attenuation and peripheral enhancement, with cystic components throughout the solid tumor usually associated with necrosis and internal hemorrhage [9]. Microscopically, SPTs feature discohesive, monomorphic cells that surround small capillaries (fibrovascular stalks) in a solid pattern. As these discohesive cells detach from the fibrovascular stalks, they form structures resembling papillae, known as pseudopapillae, and rosettes. The discohesive nature of the cells is caused by defects in β-catenin and E-cadherin. Nuclei are round to oval with irregular contours and longitudinal grooves. Pleomorphism and mitoses are infrequent [10].

The immunophenotype is variable and heterogeneous. SPTs are positive for β-catenin (100%) (both cytoplasmic and nuclear), progesterone receptors (100%), CD99 (paranuclear dot), CD10, and vimentin. These tumors express neuroendocrine markers such as CD56, neuron-specific enolase, synaptophysin, and less frequently, chromogranin A (13%). Other markers that may also be positive include cyclin D1, CD117, Friend leukemia integration 1 (Fli-1), paired-box gene 8 (Pax8), and exocrine markers. Estrogen receptors, lipase, and cytokeratins are often negative. The main differential diagnosis is the neuroendocrine tumor (NET), which also tests positive for CD56, synaptophysin, and chromogranin A. β-catenin, progesterone receptors, and CD10 are useful markers for distinguishing between these two entities [10]. As previously mentioned, the only curative treatment for these patients is the surgical resection, mostly being pancreaticoduodenectomy or distal pancreatectomy. In the case presented here, the patient had an advantage that is observed in very few cases, a very early diagnosis and a tumor confined to the pancreatic neck, so a less invasive approach, central pancreatectomy, could be performed.

Central pancreatectomy was first described by Dr. Dagradi and Dr. Serio in 1982, which is why it is also known as the Dagradi-Serio surgery. It was performed to resect and insulinoma in the pancreatic isthmus and was presented for the first time in literature in 1984 in Enciclopedia Medica Italiana. It is a surgical procedure in which a segmental resection of the pancreatic body is performed; this procedure is indicated for benign or low-grade malignant tumors located in the neck or body of the gland and measure from 2 to 5 cm that are deeply embedded in the pancreatic parenchyma and do not qualify for a enucleation [11]. The main problems associated with this procedure have been an increased risk of pancreatic fistulas with an incidence up to 54%; however, most of these heal completely with a conservative treatment alone. When the fistula originates from the pancreaticojejunostomy, the enzymes are not exposed to biliar activation; however, when the fistula is produced at the pancreaticoduodenectomy, the enzymes are exposed to activation and the conservative treatment alone is not enough. Morbidity rate ranges up to 40% and the re-operation rates are up to 18% [12]. Late complications, such as diabetes, were studied by the study of Kendall et al. at the Minneapolis Transplantation Center, where an incidence of 25% was described [13]. This can be explained by the fact that central pancreatectomy preserves almost all the body and tail, where beta cells or insulin-producing cells seem to be more numerous. Another important point needed to be discussed is the decision of waiting till after the gastric bypass surgery to perform the pancreatic surgery. A systematic review conducted showed that outcomes in obese patients are inconsistent; however, most reports have been associated with a longer surgical time, more blood loss, higher rates of pancreatic fistula, and longer length of hospital stay [14]. Which is why the decision was made to wait after the gastric bypass in order to seek to minimize the risks associated with obesity and seek the lowest possible morbidity and mortality.

## Conclusions

Incidentalomas represent a current problem associated with the wide use of imaging studies for diagnostic approaches of multiple pathologies. Although these generally represent a diagnostic challenge, in other cases, such as the one presented here, they represent a stroke of luck for patients. The early diagnosis of an SPT implies a great change in the morbidity and mortality associated with them. Something very important to highlight in this case is the fact that having an established diagnosis does not mean that the patient should be operated on immediately if it is not an emergency case and the patient is not in good physical condition presurgery. Pancreatic surgeries, especially a pancreatectomy, represent a major surgery where efforts should be made to optimize the presurgical state of health as much as possible in order to give the patient the best chance of being able to face the surgery and the challenges in the postoperative period. Here, it is also described how on many occasions biochemical changes can be observed in the postsurgical period that do not always represent a complication of the surgery but rather changes in the body associated with the trauma of the intervention. It is important to know how to define these and thus be able to identify cases that require active management and cases where close surveillance can be maintained, while the body itself processes the trauma it experienced.
